# The 5As of healthy pregnancy weight gain: possible applications in the Brazilian context to improve maternal-fetal health: Os 5As do ganho de peso saudável durante a gestação: possíveis aplicações no contexto brasileiro para melhoria da saúde materno-fetal

**DOI:** 10.1590/1516-3180.2015.02191711

**Published:** 2016-03-18

**Authors:** Danilo Fernandes da Silva, Zachary Michael Ferraro, Felipe Moretti, Helena Piccinini-Vallis, Kristi Bree Adamo

**Affiliations:** I MSc. Doctoral Student in the Department of Physical Education, Universidade Estadual de Maringá (UEM), Maringá, Paraná, Brazil.; II PhD. MD. Candidate in the Faculty of Medicine, University of Ottawa, Ottawa, Ontario, Canada.; III MD. Assistant Professor, Department of Obstetrics and Gynecology and Newborn Care, Division of Maternal-Fetal Medicine, Obstetrics and Gynecology, The Ottawa Hospital, University of Ottawa, Ottawa, Ontario, Canada.; IV MD, MSc. Assistant Professor, Department of Family Medicine, Dalhousie University, Halifax, Nova Scotia, Canada.; V PhD. Associate Professor, School of Human Kinetics, Faculty of Health Sciences, and Department of Pediatrics, Faculty of Medicine and University of Ottawa, Ottawa, Ontario, Canada.

It is well-established that women who enter pregnancy at a healthy body weight (i.e. body mass index [BMI] 18.5-24.9 kg/m^2^), and whose gestational weight gain (GWG) aligns with evidence-based guidelines, are likely to have fewer antenatal, intrapartum and postpartum complications or challenges. This will positively influence their own health as well as that of their child over the short and long-term.^1^ Excess GWG is of particular concern given that it is associated with gestational diabetes mellitus, post-partum weight retention, fetal overgrowth and, downstream, childhood obesity.^2^ The prevalence of excess GWG in Brazil has been reported to be up to 52%.^3^ Thus, there is a need to promote healthy pregnancy weight gain in an effort to optimize maternal-fetal outcomes and secure the future of public health in Brazil.

A large body of evidence supports the importance of a healthy lifestyle (i.e. healthy eating behaviour, adequate sleep, stress management, regular physical activity and limiting sedentary behaviour) during pregnancy for both mother and fetus.^4^ Despite this, there are discrepancies regarding care provider messaging and patient uptake of behavioural recommendations. Primary care providers need simple, easy-to-use frameworks that will help guide the clinical encounter in an attempt to improve dialogue with the end goal of guideline-concordant GWG. The objective of this letter is to describe a tool recently developed by the Canadian Obesity Network in an attempt to harmonize GWG approaches and support women with uptake of healthful behaviour.^5^

This practitioner guide was developed based on evidence, and is called the “5As of Healthy Pregnancy Weight Gain” (http://www.obesitynetwork.ca/pregnancy). It is a modified framework to aid primary care practitioners in helping their patients manage their GWG. The guide is rooted in motivational interviewing techniques, behaviour-change theory, and principles of patient-centeredness. Importantly, it respects the brevity of the clinical encounter during prenatal care. Briefly, the 5As are: Ask (for permission to discuss weight); Assess (the proximal and distal contextual causes of excess GWG); Advise (on risks and management options); Agree (on a feasible plan to achieve goals); and Assist (women in identifying barriers/facilitators; educate, refer and arrange follow-up).

“Ask” allows the patient to defer the discussion or to stop it from taking place at all. The point is that this is a respectful gesture on the part of the provider, who is in a situation of power. Asking permission to discuss this sensitive topic gives some power back to the patient, who, if in agreement about discussing the topic, will now actually be engaged in the discussion.

“Assess” is when the provider evaluates the guideline concordance of the weight that has been gained. However, it is also about understanding the patient: the proximal and distal contexts that can then lead to meaningful discussions. There is little point in a provider making a recommendation without an understanding of who the patient is and his or her life context.

“Advise” refers to the need to provide advice on a personalized level and in a way that considers the socioeconomic and cultural context of each woman. It is here that this framework may be a unique addition to the clinician’s tool box in Brazil. Ignoring one’s bias and making assumptions about a patient’s health-related behavior can lead to ineffective interventions and should be thoughtfully considered. For example, asking a woman to change her diet by purchasing healthy food from a vendor located far from her home when she does not have transportation may not be helpful. Here, the recommended “intervention” failed the patient, and not the other way around, so not passing judgement is key to strengthening the patient-provider relationship.

“Agree” alludes to the notion that both the expectant mother and her care team agree on a tentative (behavioral modification) plan that ultimately leads to healthy amounts of weight gain per trimester (this is based on the mother’s pre-pregnancy BMI category).

“Assist” is associated with offering education and credible resources that may help each patient to increase self-management, and this capitalizes on allied care providers when available. It is universally accepted that weight management programs are more successful when using an interdisciplinary approach. Care can be appropriately triaged, with the necessary referrals made, such that these choices are made as a team and are appropriate for each woman. Only then can unique social determinants be addressed, with minimization of complications and removal of barriers to healthier pregnancy. Much of this success also depends on arranging follow-up appointments or referrals and tracking behaviour over time.^5^

In summary, the 5As framework can be an alternative to current standard practice with the aims of harmonizing the care provider strategy and guiding healthcare professionals in their practices, while adding a personalized and empathetic approach to the patient encounter. It is strongly recommended that future studies assess the efficacy and effectiveness of the 5As framework’s ability to facilitate provider-patient dialogue and improve patient experiences. Only then can we hope to directly translate these into healthier maternal-fetal clinical outcomes (e.g., adequate GWG, less gestational diabetes and other cardiometabolic risk factors, decreased mortality, etc.). Lastly, studies focusing on translation, cultural adaption and validation of the tool are also needed. We look forward to academic discussion on this topic in order to improve the quality of the health care system.
